# Authentication of collagen VI antibodies

**DOI:** 10.1186/s13104-017-2674-x

**Published:** 2017-07-29

**Authors:** Jamie Endicott, Paul Holden, Jamie Fitzgerald

**Affiliations:** 1 Bone and Joint Center, Department of Orthopedic Surgery, Henry Ford Hospital System, Integrative Biosciences Building, 6135 Woodward Ave, Detroit, MI 48202 USA; 2 Department of Orthopaedics and Rehabilitation, Oregon Health and Science University, Portland, OR 97239 USA

**Keywords:** Collagen VI, Muscular dystrophy, Immunohistochemistry

## Abstract

**Background:**

Collagen VI is a ubiquitously-expressed macromolecule that forms unique microfibrillar assemblies in the extracellular matrix. Mutations in the *COL6A1*, *COL6A2* and *COL6A3* genes result in congenital muscular dystrophy, arguing that collagen is critical for skeletal muscle development and function. Antibodies against collagen VI are important clinical and diagnostic tools in muscular dystrophy. They are used to confirm genetic findings by detecting abnormalities in the distribution, organization and overall levels of collagen VI in cells and tissues isolated from patients.

**Methods:**

Many antibodies have been raised against tissue-purified collagen VI and individual collagen VI chains, however few have been properly validated for sensitivity and chain specificity. To address this deficiency, we compared the ability of 23 commercially-available antibodies to detect extracellular collagen VI by immunohistochemistry on frozen tissue sections. To determine chain specificity, immunoblot analyses were conducted on cell lysates isolated from cells transfected with cDNAs for each individual chain and cells expressing all three chains together.

**Results:**

Our analyses identified 15 antibodies that recognized tissue collagen VI by immunohistochemistry at varying intensities and 20 that successfully detected collagen VI by immunoblotting. Three antibodies failed to recognize collagen VI by either method under the conditions tested. All chain-specific antibodies that worked by immunoblotting specifically recognized their correct chain, and no other chains.

**Conclusions:**

This series of side-by-side comparisons reveal at least two antibodies specific for each chain that work well for immunohistochemistry on frozen sections. This validation study expands the repertoire of antibodies available for muscular dystrophy studies caused by defects in collagen VI.

**Electronic supplementary material:**

The online version of this article (doi:10.1186/s13104-017-2674-x) contains supplementary material, which is available to authorized users.

## Background

A major discussion point within the biomedical research community concerns experimental reproducibility. One recent study attempted to replicate the findings from 53 clinical experiments but found that results from just six of these studies could be independently confirmed [1]. While there are many reasons for this, a primary factor is the inadequate validation of antibodies leading to false positive (and negative) findings. Many antibodies in the public domain have been found to be poorly characterized. For example, a 2008 study found that only half of >6000 commercially-available antibodies uniquely recognized their stated targets [2]. The most common issue was poor specificity, where the antibody recognizes structurally-similar proteins in addition to the target protein. As the use of antibodies in research increases, it is critical that proper antibody authentication experiments are conducted. Funding bodies are also concerned about the apparent lack of experimental reproducibility. In the US, National Institutes of Health requires a validation plan to be provided for key research tools, including antibodies, in all grant applications from 2016 onwards (see http://grants.nih.gov/reproducibility/index.htm).

Collagen VI is present in the extracellular matrix (ECM) of skeletal muscle where it functions to anchor the basement membrane to underlying interstitial tissues. Five collagen VI chains have been described in human [3, 4]. Mutations in three of these (*COL6A1*, *COL6A2* and *COL6A3*) result in two congenital muscular dystrophies demonstrating that collagen VI is critical for muscle development. These diseases are the relatively mild Bethlem myopathy (BM) and the more severe Ullrich congenital muscular dystrophy (UCMD) [5, 6]. Studies on skeletal muscle biopsies and cultured fibroblasts isolated from affected individuals have established that, in general, the severity of disease is determined by the type of mutation. The majority of disease-causing mutations are amino acid substitutions that typically result in reduced efficiency of collagen VI assembly, leading to reduced and/or aberrant deposition of collagen VI in the muscle ECM [7–10]. Antibodies are used to assess the consequences of mutant or absent protein on collagen VI assembly by immunoblotting, and on ECM deposition and organization by immunohistochemistry [9, 11–14]. Commonly-used antibodies include VI-26 (MAB3303) raised against tissue-purified collagen VI, 3C4/Mab1944 for the α3 chain, and 6A1-H200 for α1. Public discussion within the collagen VI community about the use of antibodies for the molecular and histological analysis of collagen VI mutations [15, 16] stimulated an effort to authenticate commonly-used antibodies. In this report we define chain specificity and relative sensitivities of 23 commercially-available collagen VI antibodies by immunohistochemistry and immunoblotting.

## Methods

### Immunohistochemistry

Frozen sections of normal human skeletal muscle were obtained from the Michigan Society of Histotechnologists. Tissues were embedded in Tissue-Tek OCT (Sakura) and frozen in isopentane cooled in liquid nitrogen. Tissue blocks were stored at −80 °C before sectioning on a Microm HM550 cryostat. Transverse and longitudinal four micron-width sections were mounted on charged slides (Fisher) and stored at −80 °C before staining. Serial cryosections were warmed to room temperature and briefly fixed in acetone at −20 °C then air-dried and equilibrated in 1× PBS. Following equilibration, sections were blocked at room temperature with 10% (v/v) normal serum of the species the secondary antibody was raised in. Primary antibodies including catalogue number, species of origin, epitope (if provided) and working dilutions for each application are shown in Table 1. Working dilutions were determined via manufacturer recommended dilutions for immunohistochemistry and ranged from 1:40 to 1:400. Fluorescein isothiocyanate (FITC)-conjugated goat anti-mouse IgG (Abcam ab6785) and Cy3-conjugated goat anti-rabbit IgG diluted (Abcam ab6939) secondary antibodies were used for the majority of the antibody panel. In the case of primary antibody H3-2, a mouse IgM, FITC-conjugated goat anti-mouse IgM diluted (Abcam ab97229) secondary antibody was used. For primary antibodies raised in goat, a Cy2 donkey anti-goat secondary was used (GeneTex GTX26948). Cryosections were incubated with primary and secondary antibodies for 1 h at room temperature. Sections were washed in 1× PBS between primary and secondary incubations and stained sections were mounted with ProLong Gold Antifade with DAPI (Cell Signaling Technology) (UV/nuclear staining not shown). All immunofluorescence images were obtained via Photometrics camera (CoolSNAP MYO) paired with Nikon Eclipse T*i*-S fluorescence microscope. Images were analyzed with NIS-Elements software.

**Table 1:** Summary of collagen VI antibodies and experimental conditions

Antibody	Supplier	Cat number	Species	Immunogen	IHC dilution	IB dilution
Collagen VI VI-26	Millipore	MAB3303	mou mAb	Human-tissue not specified	1:100	1:500
Collagen VI	Fitzgerald Ind.	70R-CR009X	rab pAb	Human/bovine placenta	1:100	1:1000
Collagen VI 172C2	Santa Cruz	sc-47764	mou mAb	Human-tissue not specified	1:100	1:500
Collagen VI 5C6	U. Iowa DSHB	–	mou mAb	Human fetal membranes	1:100	1:500
Collagen VI	Millipore	AB7821	rab pAb	Human placenta	1:100	1:500
COL6A1 H200	Santa Cruz	sc-20649	rab pAb	α1 chain, amino acid 51-250	1:40	1:500
COL6A1	Abcam	ab199720	rab mAb	α1 chain, within aa 800 to C-term.	1:400	1:2000
COL6A1	Abcam	ab182744	rab mAb	α1 chain within amino acid 1-250	1:100	1:1000
COL6A1	Proteintech Gp	17023-1-AP	rab pAb	Not available	1:100	1:500
COL6A1 B4	Santa Cruz	sc-377143	mou mAb	α1 chain amino acid 53-86	1:40	1:500
COL6A2	Proteintech Gp	14853-1-AP	rab pAb	Not available	1:100	1:500
COL6A2 EPR7889	Abcam	ab180855	rab mAb	α2 chain, amino acid 800-900	1:400	1:1000
COL6A2	Abcam	ab172606	rab mAb	α2 chain, amino acid 200-300	1:100	1:1000
COL6A2 D20	Santa Cruz	sc-83607	rab pAb	α2 chain, internal epitope	1:40	1:500
COL6A2 H300	Santa Cruz	sc-292186	rab pAb	α2 chain, amino acid 241-540	1:40	1:500
COL6A2 K15	Santa Cruz	sc-377143	rab pAb	α2 chain, C-terminus	1:40	1:500
COL6A2 B7	Santa Cruz	sc-374566	mou mAb	α2 chain, amino acid 241-540	1:40	1:500
COL6A3 G18	Santa Cruz	sc-131139	goat pAb	Not available	1:40	1:500
COL6A3 3C4	Millipore	MAB1944	mou mAb	Not available	1:100	1:1000
COL6A3 3C4	Santa Cruz	sc-47712	mou mAb	Not available	1:100	1:250
COL6A3 H300	Santa Cruz	sc-367543	rab pAb	α3 chain, amino acid 301-600	1:40	1:500
COL6A3 H3-2	Santa Cruz	sc-81766	mou mAb	α3 chain, globular domains	1:100	1:500
COL6A3 64C11	Abcam	ab49273	mou mAb	α3 chain, 295 aa C-term. frag	1:100	1:1000
COL6A3 N12	Santa Cruz	sc-131140	goat pAb	Not available	1:40	1:500

Antibodies are grouped by name and clone and each entry includes supplier and catalogue number, species antibody was raised in and whether the antibody is monoclonal or polyclonal. Also listed is the immunogen/epitope used to generate the antibody, if known, and the dilutions used for each application

### Cell transfections

cDNAs for each chain were PCR amplified from existing cDNA clones and subcloned into a mammalian expression vector (pCDNA6). COL6A1 and COL6A2 were full-length cDNA clones encompassing the N1 to C2 domains. The COL6A3 cDNA spanned the N6 to C5 domains and we have previously established that this cDNA assembles into multimeric collagen VI [17]. All clones were verified by Sanger sequencing.

HEK-293 cells (ATCC, CRL-1573) were independently transfected with cDNAs for the human *COL6A1*, *COL6A2* and *COL6A3* genes producing four cell lines that stably express the α1, α2 and α3 chains of collagen VI separately, and one line that expresses all three chains together. We have previously established that HEK-293 cells do not express detectable levels of *COL6A1*, *COL6A2* or *COL6A3* mRNA by RT-PCR (data not shown).

### Immunostaining transfected cells

HEK-293 cultures expressing each chain individually were grown to confluency in Dulbecco’s modified Eagles medium (GIBCO) supplemented with 10% fetal calf serum (GIBCO) in 4-well chamber slides. Cells were incubated overnight in media supplemented with 0.25 mM sodium ascorbate to facilitate collagen biosynthesis. Cells were fixed in 2% paraformaldehyde, permeabilized with 0.1% v/v Triton-X100 (Sigma), and blocked with 10% normal goat serum (Abcam, ab156046) in 1× PBS. Primary and secondary antibodies were added at the same concentration and incubation times as for the immunohistochemical studies (see Table 1).

### Cell lysate preparation

Transfected HEK-293 cells were grown to confluence in DMEM with 10% (v/v) fetal calf serum, then supplemented with 0.25 mM sodium ascorbate for 24 h. Media was removed and the cells gently washed with 1× PBS to remove residual media. Lysates were prepared by scraping the adherent cell layer into 1% Nonidet-P40 (Sigma) in 1× PBS with EDTA-free protease inhibitor cocktail (Roche). Lysates were incubated under agitation for 24 h at 4 °C, centrifuged to pellet insoluble material, and the supernatants collected. To ensure equal gel loading, sample protein concentration were determined using a BCA assay kit (Thermo Scientific) and aliquots were resolved under reducing (20 mM DTT) and non-reducing conditions by SDS-PAGE. Gels were either 6% (w/v) polyacrylamide made in-house or 4–15% (w/v) polyacrylamide gradient gels purchased from Bio-Rad.

### Immunoblotting

For immunoblotting, collagen chains resolved by SDS-PAGE were transferred onto PVDF membranes (Millipore), then blocked for 1 h at room-temperature in 4% nonfat dried milk in 0.1% PBS/tween-20. Blots were probed individually with collagen VI antisera, some chain-specific and some raised against tissue-purified collagen VI. Antibody dilutions are listed in Table 1. Bound primary antibody was detected with either anti-goat, -rabbit, or—mouse fluorescently-labeled secondary IgG. Blots were visualized on the Odyssey CLx imager (LiCor).

All immunoblot and immunohistochemistry experiments were repeated at least once to confirm results.

## Results

To assess the relative efficiency of collagen VI antibodies to detect collagen VI in muscle, cross and longitudinal frozen sections of normal human skeletal muscle were immunostained with a panel of 23 commercially-available collagen VI antibodies. Primary antibodies were detected by fluorescently-labelled secondary antibodies. Experimental conditions were identical except for antibody dilutions, which were selected based on manufacturers guidelines, and choice of secondary antibody which was dictated by the species the primary antibody was raised in.

To validate the collagen VI antibodies for use in immunoblot studies, we developed four HEK-293 fibroblast cell lines; three that express α1, α2 and α3 individually and one that expresses all three together. The commonly accepted dogma is that in cells that endogenously produce collagen VI, the three chains co-assemble into heterotrimers in a 1:1:1 stoichiometric ratio [18, 19]. We assume that the collagen VI produced by the triple-transfected cells assemble in accordance with this dogma, although other assemblies may be possible. Using this panel it is possible to determine, (1) whether the antibodies recognize the correct individual collagen VI chains and/or multimeric collagen VI, (2) whether they cross-react with other closely-related collagen VI chains and (3), their relative sensitivities in detecting the same α chain. A summary of results from the immunohistochemical and immunoblot experiments is presented in Table 2.

**Table 2:** Summary of immunohistochemistry and immunoblot results

Antibody	Cat number	Signal by IHC	α1 lysate	α2 lysate	α3 lysate	α1 α2 α3 lysate	Figures
COL6A1 H200	sc-20649	++	++ (R)	−	−	+	1
COL6A1	ab199720	+++	+++ (R)/+++ (NR)	−	−	+	1
COL6A1	ab182744	++	++ (R)	−	−	+	1
COL6A1	17023-1-AP	+++	+++ (R)/+++ (NR)	−	−	+	1
COL6A1 B4	sc-377143	−	+++ (R)	−	−	+	1
COL6A2	14853-1-AP	++	−	++ (R)/+ (NR)	−	nd	2
COL6A2 EPR7889	ab180855	+++	−	+++ (R)	−	−	2
COL6A2	ab172606	++	−	+ (R)	−	nd	2
COL6A2 D20	sc-83607	+++	−	+++ (R)	−	++	2
COL6A2 H300	sc-292186	++	−	+ (R)	−	+	2
COL6A2 K15	sc-377143	+++	−	+ (R)	−	+	2
COL6A2 B7	sc-374566	−	−	+++ (R)/+ (NR)	−	++	2
COL6A3 G18	sc-131139	++	−	−	+ (NR)	+	3
COL6A3 3C4	MAB1944	+++	−	−	+ (R)/++ (NR)	−	3
COL6A3 3C4	sc-47712		−	−	+	nd	3
COL6A3 H300	sc-367543	+++	−	−	−	−	3
COL6A3 H3-2	sc-81766	−	−	−	−	−	3
COL6A3 64C11	ab49273	−	−	−	−	−	3
COL6A3 N12	sc-131140	−	−	−	−	nd	3
Collagen VI VI-26	MAB3303	+++	−	−	−	+	4
Collagen VI	70R-CR009X	+++	−	−	++ (NR)	+++	4
Collagen VI 172C2	sc-47764	−	−	−	++ (NR)	−	4
Collagen VI 5C6		−	−	−	−	+	4
Collagen VI	AB7821	−	−	Non-sp.	Non-sp.	++	4

Each antibody is scored for signal intensity on immunohistochemical signal and, for immunoblots, band intensity ranging from ‘−’ representing no detectable signal, to ‘+++’ representing strong signal. The description of immunoblots signal strength scores reduced (R) and non-reduced (NR) samples separately. Some blots on triple transfected lysates were not conducted and are designated as not determined (nd)

Chain specificity was also confirmed for three antibodies by immunocytochemistry on transfected cells (Additional file 1: Figure S1). These antibodies were; ab199720 for α1, ab180855 for α2 and 70R-CR009X for α3. While these cells do not produce a collagen VI extracellular matrix because they lack the other chains necessary for heterotrimeric assembly, they are used in the current study to confirm chain specificity as indicated by immunoblot data.

### Antibodies raised against tissue-purified collagen VI

**Fig. 1:**
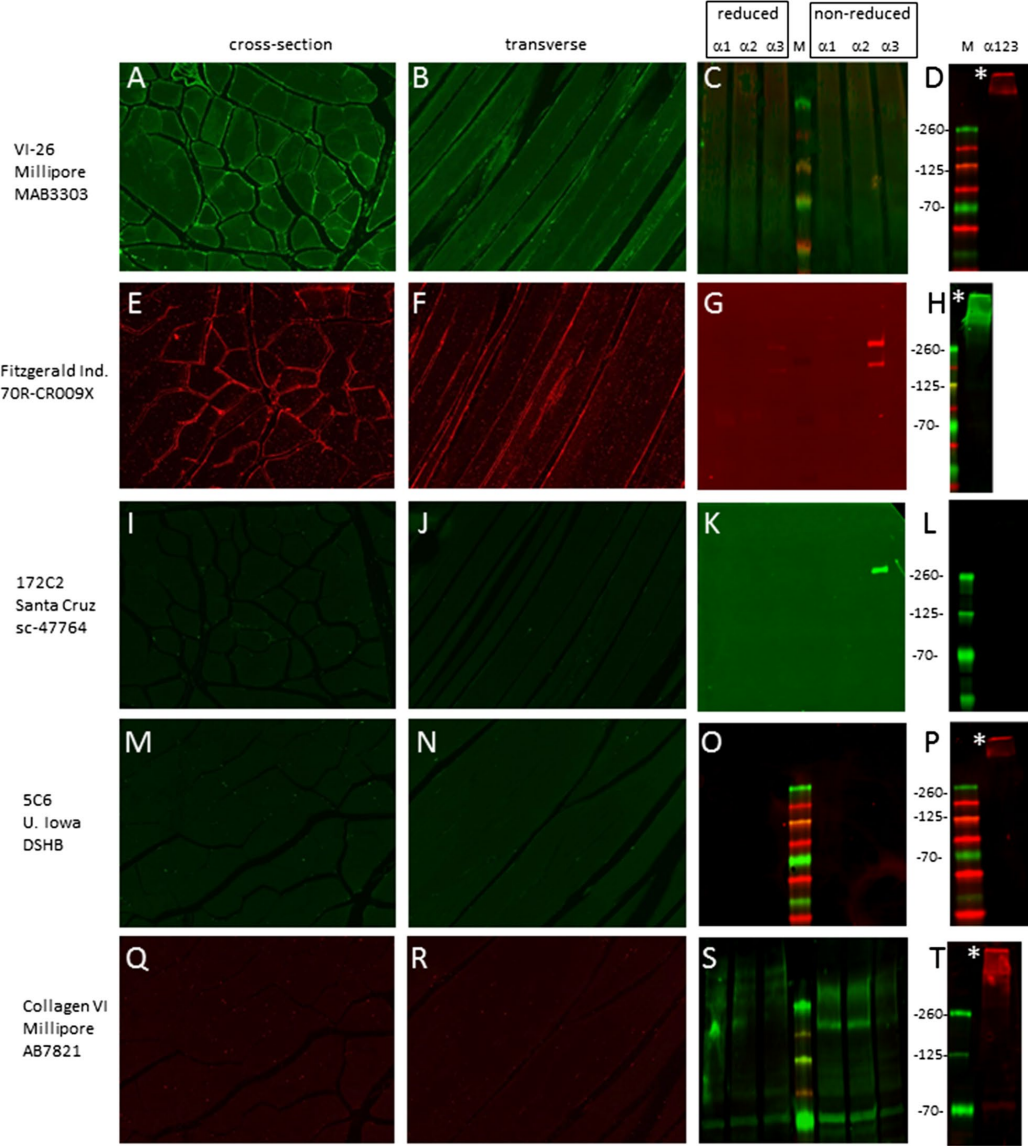
Validation of antisera raised against tissue-purified collagen VI. Antibody clone, supplier and catalogue number are listed down the *left-hand side*. Staining was conducted on cross- (**A**, **E**, **I**, **M**, **Q**) and transverse (**B**, **F**, **J**, **N** and **R**) sections of normal human skeletal muscle using dilutions described in Table 1. Blots containing lysates from cells expressing α1, α2 and α3 chains run under reducing (*boxed first three lanes*) and non-reducing (*boxed second set of lanes*) conditions are shown in **C**, **G**, **K**, **O** and **S**. *Each blot* was probed with antibodies indicated on *left* at the dilutions shown in Table 1. Blots from cell lysates (non-reduced) expressing multimeric collagen VI expressing all three chains were also probed (**D**, **H**, **L**, **P** and **T**). *Asterisks* denote high molecular weight collagen VI band. Migration position of molecular weight markers (in kDa) is shown between each pair of immunoblots

Five antibodies that were raised against collagen VI from pepsin-treated tissue were tested (Fig. 1). Of these, only two showed detectable signals by immunohistochemistry: monoclonal MAB3303 (clone VI-26 from Millipore) (panels A and B) and polyclonal 70R-CR009X (Fitzgerald Industries) (E and F). The staining was clearly pericellular in cross- and longitudinal sections, as expected for collagen VI. In addition, both antibodies recognized high molecular weight collagen VI in lysates from cells transfected with all three chains (D and H). Interestingly, the Fitzgerald Industries antibody also recognized individual α3 chains when electrophoresed under non-reducing conditions suggesting that the α3 chain may be the major epitope for this antibody (G). This finding was confirmed in transfected cells where 70R-CR009X stained cells expressing α3 chains but not those expressing α1 nor α2 chains (Additional file 1: Figure S1, panels C, H and M). This is surprising since native collagen VI was used as the immunogen and may suggest that α3 chains are ‘preferentially’ exposed on native collagen VI fibrils. Alternatively, the α3 chain may have a more immunogenic structure than α1 and α2 chains in native collagen VI. Of the antibodies that failed to stain tissue, 5C6 is of interest because it has been used previously in immuno-gold labeling experiments to detect collagen VI in skin and cartilage [20, 21] where presumably collagen VI structures are in their native form. It is unclear why 5C6 failed to stain muscle frozen tissue (M and N) but the 5C6 epitope may not be revealed by the fixation and section preparation used in this study, or alternatively, skin and cartilage collagen VI has a different supramolecular architecture than muscle collagen VI and epitope accessibility varies between the two tissues. The sc-47764 antibody (clone 172C2 from Santa Cruz) did not detect muscle collagen VI by immunohistochemistry (I and J) but recognized individual α3 chains by blotting (K). The Millipore antibody AB7821 did not detect collagen VI by immunohistochemistry and reacted with multiple bands by immunoblotting (Q-T).

### Antibodies directed against individual collagen VI chains

#### α1(VI)

**Fig. 2:**
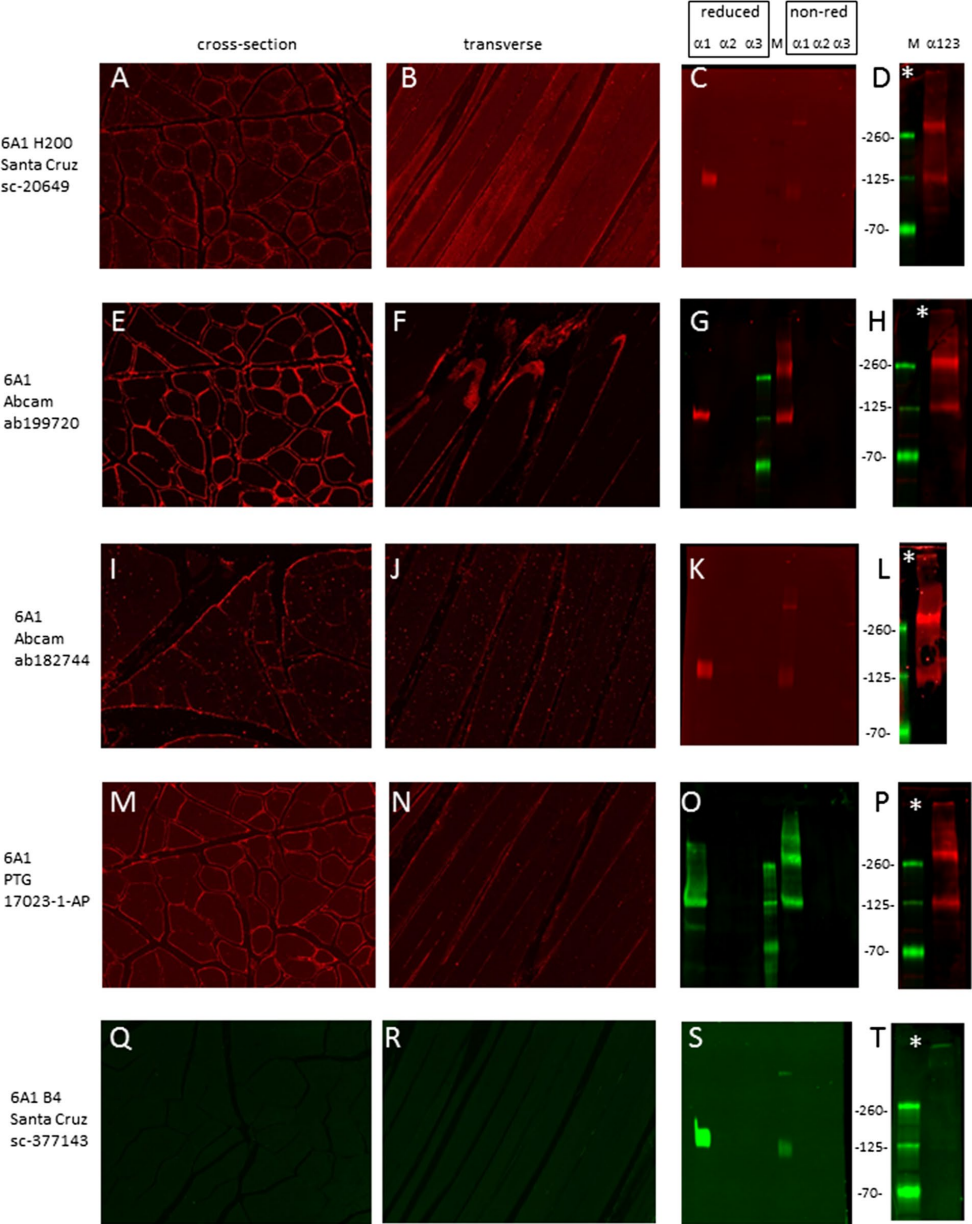
Detection of the α1 chain of collagen VI. Immunostaining was conducted on cross- (**A**, **E**, **I**, **M**, **Q**) and transverse (**B**, **F**, **J**, **N** and **R**) sections of normal human skeletal muscle. Antibodies are listed on *left*. Blots containing reduced and non-reduced sets of lysates from cells expressing α1, α2 and α3 chains individually (**C**, **G**, **K**, **O** and **S**) were probed with each α1 antibody. Blots containing lysates from cells expressing multimeric collagen VI containing all three chains were resolved under non-reducing conditions and probed (**D**, **H**, **L**, **P** and **T**). Migration position of molecular weight markers (in kDa) is shown between each pair of immunoblots

Four of the five antibodies against the α1 chain produced a signal on skeletal muscle sections, with ab199720 (abcam) (Fig. 2, panels E, F) and 17023-1-AP (ProteinTech Group) (M and N) detecting highest levels in the pericellular space. The sc-20649 (clone H200 from Santa Cruz) staining intensity was weaker and intracellular structures appeared to be positive (A and B). Chain specificity immunoblots revealed that all five anti- α1 antibodies tested recognized α1 chains, and no other chains, when resolved under reducing conditions at the predicted molecular weight of 140 kDa (C, G, K, O and S). Antibodies 17023-1-AP and ab199720 recognized α1 chains in both reduced and non-reduced samples (G and O). Interestingly, both these antibodies revealed the presence of discrete α1 oligomers larger than 140 kDa suggesting that α1-chains may undergo self-assembly to form homo-oligomers in these cells. Four antibodies recognized α1 chains at approximately 180 kDa in lysate containing all three chains which corresponds to the size of α1 dimers (D, H, L and P). All α1 antibodies recognize a small amount of high-molecular weight collagen VI near the top of the blots.

In transfected cells, ab199720 gave a strong signal in cultures of cells expressing α1, but not in cells expressing α2 or α3 chains (Additional file 1: Figure S1, panels A, F and K). This confirms the immunoblot finding (see Fig. 2G) that this antibody specifically recognizes the α1 chain.

#### α2(VI)

**Fig. 3:**
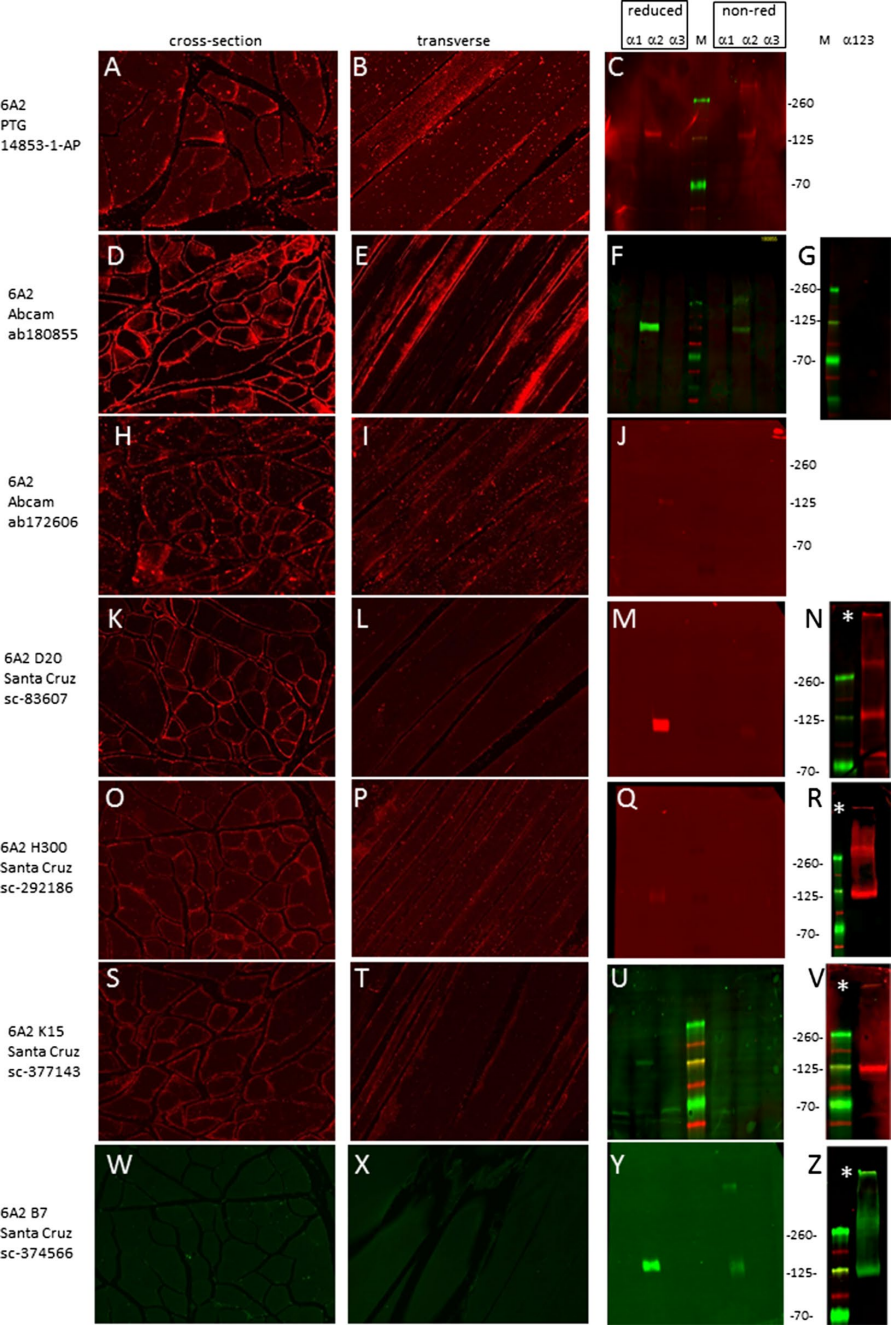
Detection of the α2 chain. Immunostaining was conducted on cross- (**A**, **D**, **H**, **K**, **O**, **S** and **W**) and transverse (**B**, **E**, **I**, **L**, **P**, **T** and **X**) sections of skeletal muscle. Blots containing reduced and non-reduced sets of lysates from cells expressing α1, α2 and α3 chains individually (**C**, **F**, **J**, **M**, **Q**, **U** and **Y**) were probed with each α2 antibody. Blots containing multimeric collagen VI expressing all three chains and electrophoresed under non-reducing conditions were probed (**G**, **N**, **R**, **V** and **Z**). Migration position of molecular weight markers (in kDa) is shown

Six out of seven α2-antisera stained skeletal muscle, with ab180855 (abcam) demonstrating the strongest signal (Fig. 3, panel D). Only sc-374566 (clone B7 from Santa Cruz) failed to detect muscle collagen VI (W and X). All seven α2 antibodies recognized their epitopes when lysates were resolved under reducing conditions although with a range of sensitivities. Three α2 antisera worked well (F, M and Y) with clearly detectable bands at the correct molecular weight (140 kDa) and four performed poorly with only faint α2 bands present (C, J, Q and U). Only sc-374566 recognized individual non-reduced α2 chains, albeit weakly (Y). Several antibodies recognized multimeric collagen VI with sc-374566, sc-83607 (clone D20 from Santa Cruz), and sc-377143 (clone K15 from Santa Cruz) (asterisks in panels N, V and Z) showing the strongest signals. ab180855 stained cells expressing α2 but not α1 nor α3 (Additional file 1: Figure S1, panels B, G and L) confirming immunoblot findings for this antibody (Fig. 3F).

#### α3(VI)

**Fig. 4:**
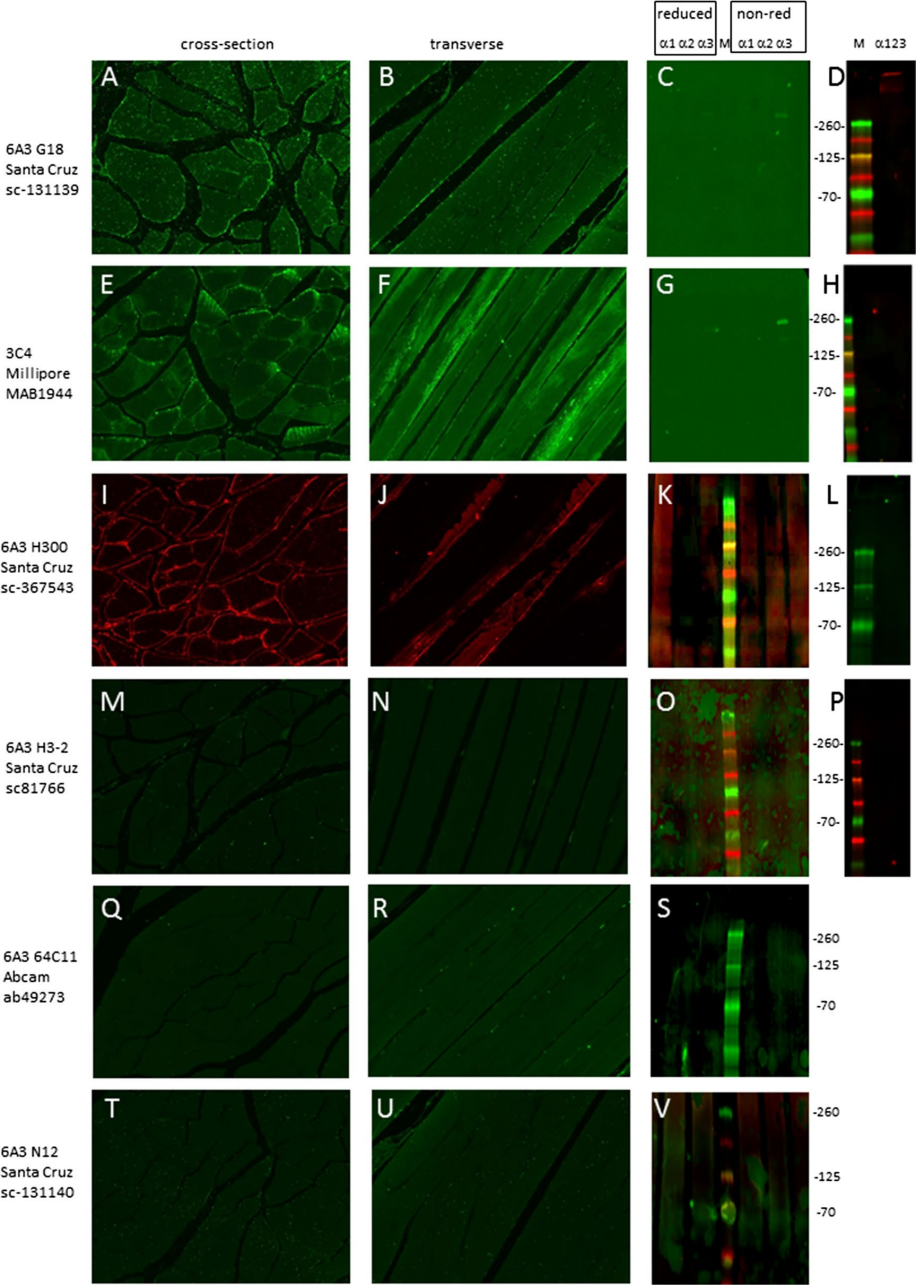
Detection of α3. Immunostaining was conducted on cross- (**A**, **E**, **I**, **M**, **Q** and **T**) and transverse (**B**, **F**, **J**, **N**, **R** and **U**) sections of skeletal muscle. Blots containing reduced and non-reduced sets of lysates from cells expressing α1, α2 and α3 chains individually (**C**, **G**, **K**, **O**, **S** and **V**) were probed for α3. Blots from cell expressing multimeric collagen VI expressing all three chains resolved under non-reducing conditions were probed (**D**, **H**, **L**, and **P**). Migration position of molecular weight markers (in kDa) is shown between each pair of immunoblots

Three of six α3 antibodies stained skeletal muscle including the commonly used 3C4/MAB1944 (Millipore) which also appeared to stain intracellular structures (Fig. 4 panels A, B, E, F, I and J). 3C4/MAB1944 and sc-131139 (clone G18) recognized their 260 kDa target under non-reducing conditions [22, 23] (C and G). The remaining α3 antibodies failed to recognize trimeric collagen VI nor individual chains under reducing or non-reducing conditions. Three antisera; sc-81766 (clone H3-2 from Santa Cruz), ab49273 (clone 64C11 from Abcam) and sc-131140 (clone N12 from Santa Cruz) failed to recognize collagen VI by either method using the experimental conditions used in this study (M to V).

**Fig. 5:**
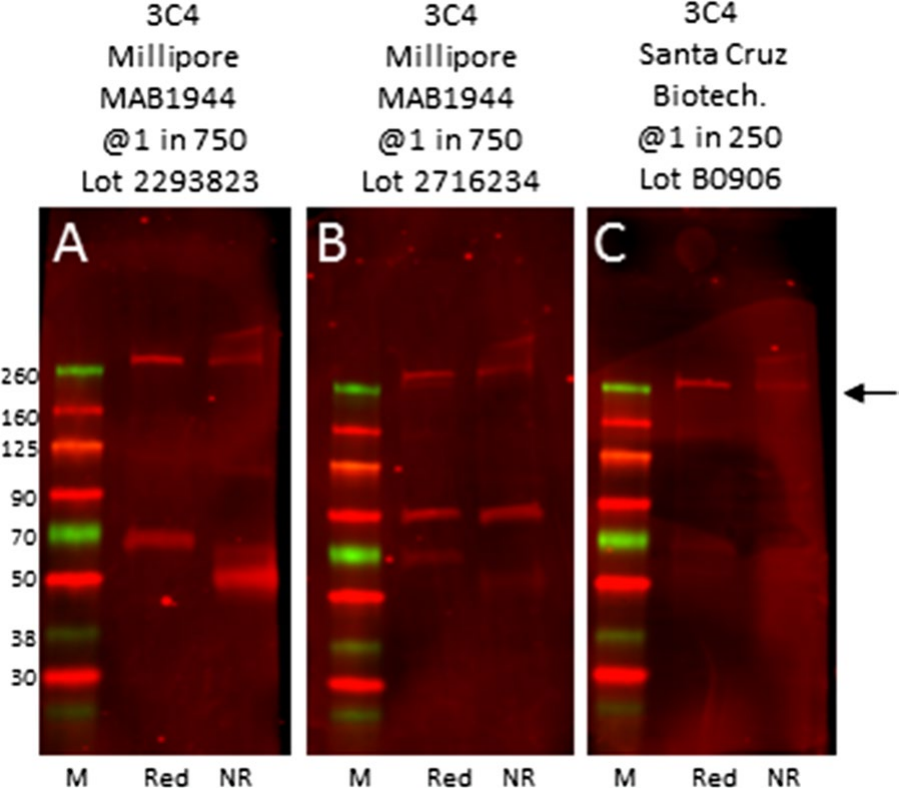
Comparison of 3C4/MAB1944 antisera. Lysates from HEK-293 cells transfected with COL6A3 cDNA were immunoblotted using two different lots of MAB1944 from Millipore (**A**, **B**) and 3C4 from Santa Cruz (**C**) under reducing (*Red*) and non-reducing conditions (NR). Molecular weight markers, in kDa, are on *left*. *Arrow* showing migration position of α3 chain is shown on *right*

In the course of these studies we became aware that the mouse monoclonal antibody 3C4/MAB1944, which recognizes the α3(VI) chain, gave different results depending on supplier and batch number (Fig. 5). As shown in Fig. 5C, 3C4 from Santa Cruz recognized α3(VI) at the correct molecular weight of 260 kDa when resolved under reducing conditions. In contrast, the same antibody clone from Millipore recognized α3 at 260 kDa and additional bands at 50, 70 and 90 kDa depending on lot number (Fig. 5A, B). 3C4 is widely used in studies on human muscle using immunohistochemistry. Our data suggest that care should be taken in interpreting data using these batches of antibody from Millipore.

#### Controls

Control experiments where sections were stained using rabbit, mouse and goat IgGs at dilutions of 1:100 did not detect any collagen VI (Additional file 2: Figure S2, panels A–F). Similarly, blots containing lysates from triple transfected cells probed with mouse IgG and normal rabbit and goat serum failed to detect significant bands (G–I). A faint band at 70 kDa is present on the blot probed with normal rabbit serum but this is smaller than the 140 kDa α1 and α2 bands and 260 kDa α3 bands and represents background (asterisk in panel H).

## Discussion

To provide a meaningful assessment of the relative usefulness of collagen VI antibodies for immunohistochemistry 23 collagen VI antibodies from Abcam, Santa Cruz, ProteinTech Group and Millipore were tested for reactivity on human muscle sections under standard conditions. Since aberrant collagen VI tissue deposition can be caused by mutations in three genetically-distinct collagen VI chains and it would be valuable to know whether any antibody cross-reacted with the other structurally-similar chains, each antibody was tested for chain specificity by immunoblot analyses and, for three antibodies, immunocytochemistry. Comparative testing revealed that 15 out of 23 antibodies detected pericellular collagen VI on human skeletal muscle sections. The best antibodies specific for each chain are: ab199720 and 17023-1-AP for α1, ab180855 and 14853-1-AP for α2, 3C4/MAB1944 and sc-367543 for α3 (see Table 2). The only two antibodies raised against tissue collagen VI that detected multimeric collagen VI by immunocytochemistry were MAB3033 and 70R-CR009x.

14 out of 18 chain-specific antibodies recognized their target collagen VI chains although with a wide range of sensitivities. Notably, all 14 antibodies demonstrated chain specificity with each antisera recognizing a single collagen VI chain and none appeared to cross-react with the other collagen VI chains. However, good specificity within the collagen VI family does not exclude the possibility that these antibodies bind to other collagens or non-collagenous proteins. Each distinct chain was recognized by at least two different antibodies with high sensitivity (Table 2). The finding that antisera against α1 and α2 primarily recognizes the reduced form of the chain suggests that these chains form intra- or inter-chain disulfide bonds that nominally mask the epitopes, and complete reduction of disulfides is essential for optimal antibody recognition. Epitopes within high molecular weight collagen VI were detected by multiple antibodies with the three strongest signals being ab182744 against α1, and sc-374566 and sc-83607 against α2.

There has been discussion in the collagen VI literature regarding the use of VI-26/MAB3303 and 3C4 for immunostaining patient muscle tissue and fibroblasts [15, 16]. It has been reported that VI-26 is less sensitive at detecting collagen VI in muscle and fibroblast preparations compared to 3C4 leading to an underestimation of the amount of collagen VI degradation [23]. Our data indicates that they recognize different forms of collagen VI; VI-26 detects high-molecular weight, (presumably) triple helical collagen VI but not any individual chains (Fig. 1C, D) whereas 3C4 detects the α3 chain and not collagen VI multimers (Fig. 4G, H). These antibodies clearly recognize different epitopes and may detect different sub-populations of collagen VI fibrils in the ECM. We suggest staining using more than one antibody when detecting collagen VI by immunohistochemistry.

## Conclusions

This study expands the list of collagen VI antibodies that can be used in immunoblotting for the analysis of collagen VI on patient samples. Antibodies with high specificity and sensitivity are available for each collagen VI chain. Most α1 and α2 antisera perform best on reduced material; in contrast the majority of α3 antisera tested performed better on non-reduced samples. Several antibodies recognize multimeric collagen VI with the strongest signal from the 70R-CR009X antibody.

There have been calls to standardize antibody production, characterization and use [24, 25]. It is recommended that journals insist that authors be more transparent and consistent with reporting antibody details [26]. We echo this sentiment and reiterate the need to include basic information when reporting antibody experiments by including dilutions used, vendor and catalogue/clone numbers, and species antibody was raised in. In addition, it is essential to include controls such as isotype controls for monoclonal antibodies, pre-immune serum controls for polyclonal antisera and blocking peptide controls for peptide antibodies, if possible.

## Additional files

**Additional file 1: Figure S1.** Immunostaining transfected cells for antibody specificity. HEK-293 cells transfected with cDNAs for COL6A1 (panels A-E), COL6A2 (F-J) and COL6A3 (K-O) were stained for α1 chains (A, F and K), α2 chains (B, G and L) and collagen VI (C, H and M). Negative control stains for rabbit (D, I and N) and mouse (E, J and O) IgGs are shown. Each antibody recognizes its correct chain and not the other chains.

**Additional file 2: Figure S2.** Experimental controls for immunohistochemical and immunoblot experiments. Normal human skeletal muscle cross- and transverse sections were stained for rabbit (panels A and B), mouse (C and D) and goat (E and F) IgGs were used at a dilution of 1 in 100 and detected using the indicated fluorescently-labelled secondary antibodies. All controls were blank. Blots containing lysates from HEK-293 cells transfected with all three chains were probed for mouse IgG (panel G), normal rabbit (H) or normal goat (I) serum. Migration positions of molecular weight markers are shown on left. A faint band at 70-75 kDa is present in panel H (indicated by asterisk).
