# Supplementary figures and images for: Authentication of collagen VI antibodies
Source: BMC Res Notes. 2017 Jul 29;10:358. doi: 10.1186/s13104-017-2674-x (PMC5534245; doi:10.1186/s13104-017-2674-x)

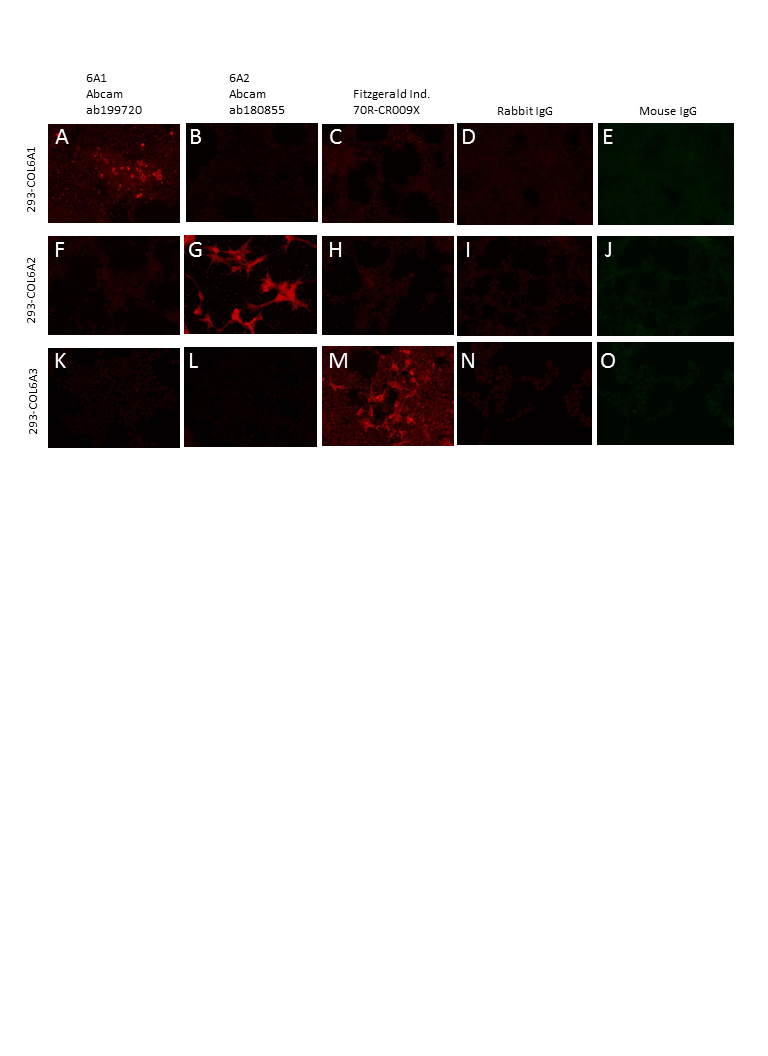

Supplement: Supplementary file 1 — Additional file 1: Figure S1. Immunostaining transfected cells for antibody specificity. HEK-293 cells transfected with cDNAs for COL6A1 (panels A-E), COL6A2 (F-J) and COL6A3 (K-O) were stained for α1 chains (A, F and K), α2 chains (B, G and L) and collagen VI (C, H and M). Negative control stains for rabbit (D, I and N) and mouse (E, J and O) IgGs are shown. Each antibody recognizes its correct chain and not the other chains. [file 13104_2017_2674_MOESM1_ESM.tif]

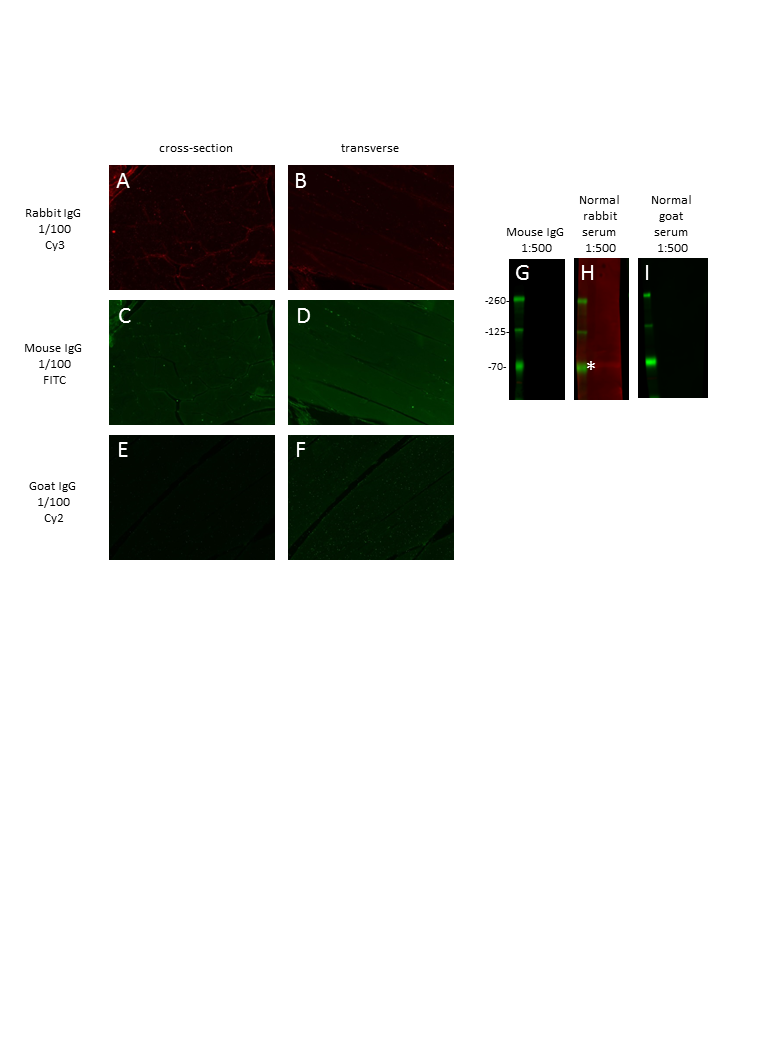

Supplement: Supplementary file 2 — Additional file 2: Figure S2. Experimental controls for immunohistochemical and immunoblot experiments. Normal human skeletal muscle cross- and transverse sections were stained for rabbit (panels A and B), mouse (C and D) and goat (E and F) IgGs were used at a dilution of 1 in 100 and detected using the indicated fluorescently-labelled secondary antibodies. All controls were blank. Blots containing lysates from HEK-293 cells transfected with all three chains were probed for mouse IgG (panel G), normal rabbit (H) or normal goat (I) serum. Migration positions of molecular weight markers are shown on left. A faint band at 70-75 kDa is present in panel H (indicated by asterisk). [file 13104_2017_2674_MOESM2_ESM.tif]
